# Mesoporous Ternary Nitrides of Earth-Abundant Metals as Oxygen Evolution Electrocatalyst

**DOI:** 10.1007/s40820-020-0412-8

**Published:** 2020-03-26

**Authors:** Ali Saad, Hangjia Shen, Zhixing Cheng, Ramis Arbi, Beibei Guo, Lok Shu Hui, Kunyu Liang, Siqi Liu, John Paul Attfield, Ayse Turak, Jiacheng Wang, Minghui Yang

**Affiliations:** 1 Ningbo Institute of Materials Technology and Engineering, Chinese Academy of Sciences, Ningbo, 315201 Zhejiang Province, People’s Republic of China; 2 Center of Materials Science and Optoelectronics Engineering, University of Chinese Academy of Sciences, Beijing, 100049 People’s Republic of China; 3 Department of Engineering Physics, McMaster University, Hamilton, L8S 4L7 Canada; 4 State Key Laboratory of High Performance Ceramics and Superfine Microstructure, Shanghai Institute of Ceramics, Chinese Academy of Sciences, 1295 Dingxi Road, Shanghai, 200050 People’s Republic of China; 5 Centre for Science at Extreme Conditions and EaStCHEM School of Chemistry, University of Edinburgh, Kings Buildings, West Mains Road, Edinburgh, EH9 3JJ UK

**Keywords:** Ordered mesoporous structure, Hard template, Ni_3_FeN, Oxygen evolution reaction, Zn–air battery

## Abstract

As sustainable energy becomes a major concern for modern society, renewable and clean energy systems need highly active, stable, and low-cost catalysts for the oxygen evolution reaction (OER). Mesoporous materials offer an attractive route for generating efficient electrocatalysts with high mass transport capabilities. Herein, we report an efficient hard templating pathway to design and synthesize three-dimensional (3-D) mesoporous ternary nickel iron nitride (Ni3FeN). The as-synthesized electrocatalyst shows good OER performance in an alkaline solution with low overpotential (259 mV) and a small Tafel slope (54 mV dec^−1^), giving superior performance to IrO_2_ and RuO_2_ catalysts. The highly active contact area, the hierarchical porosity, and the synergistic effect of bimetal atoms contributed to the improved electrocatalytic performance toward OER. In a practical rechargeable Zn–air battery, mesoporous Ni_3_FeN is also shown to deliver a lower charging voltage and longer lifetime than RuO_2_. This work opens up a new promising approach to synthesize active OER electrocatalysts for energy-related devices.

**Electronic supplementary material:**

The online version of this article (10.1007/s40820-020-0412-8) contains supplementary material, which is available to authorized users.

## Highlights

- 3D mesoporous Ni_3_FeN was constructed through hard templating and thermal nitridation.
- Ni_3_FeN exhibits superior electrochemical performance for OER with a small overpotential of 259 mV to achieve a 10 mA cm^−2^.
- Ni_3_FeN can also deliver a lower charging voltage and longer lifetime than RuO_2_ in a rechargeable Zn–air battery.

**Electronic supplementary material:**

The online version of this article (10.1007/s40820-020-0412-8) contains supplementary material, which is available to authorized users.

## Graphical abstract

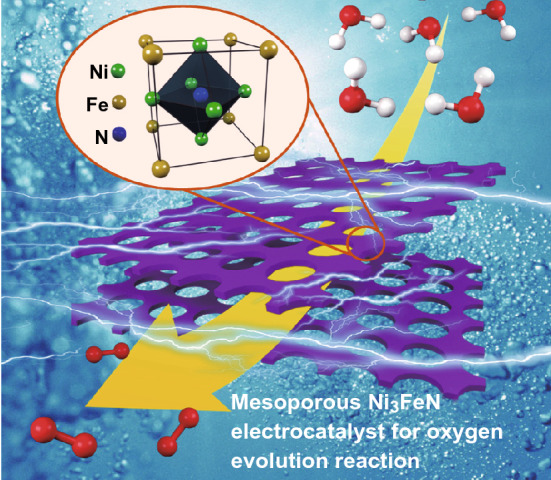

## Introduction

Among the electrochemical processes, the electrocatalytic oxygen evolution reaction (OER) has considerable scientific and economic advantages for energy conversion and storage applications, such as water splitting or metal–air batteries [1, 2]. Particularly, the small overpotentials and fast kinetics required for catalyzing OER make it potentially cost-effective. Using precious metal catalysts ruthenium (Ru) and iridium (Ir) oxides, the potential of this technology has been proved with excellent electrocatalytic characteristics [3, 4]. However, these noble metal-based catalysts are impractical due to their rarity and high cost, limiting their widespread use [5]. To address this concern, efforts have been made toward developing earth-abundant low-cost and highly active OER electrode materials, such as metal-perovskite oxides [6], sulfides [7], nitrides [8], and phosphides [9]. Among them, transition metal nitrides (TMNs) have emerged as promising low-cost alternatives due to their rapid electron transport properties and excellent chemical stability [10].

A significant decrease in overpotential can be achieved through various approaches to optimize the surface nanostructure and electronic states for OER electrocatalysts [11, 12]. One approach, the introduction of an additional transition metal element, allows tuning of the electronic structure and improvement of the precipitation energies compared to single-metal materials [13] due to the synergistic effect of a mixed metal system. For example, the incorporation of Fe into CoN has been shown to improve the catalytic performance compared to pure cobalt nitride [14, 15]. As another approach, the formation of nanostructured TMNs can provide abundant accessible active sites, high infiltration capacity, and large contact area [16]. By combining these two approaches, three-dimensional ordered mesoporous mixed metal nitrides could be made with uniform mesochannels, large surface areas and pore volume, and high electron conductivity. However, they would only be of practical interest in OER electrocatalysis [17, 18]. There has been some success in producing mixed metal transition metal nitrides by, for example, heating the respective oxide precursor under ammonia [14, 16], plasma-based nitridation [19], or in situ nitridation of metals supported on nitrogen-doped carbon [20]. However, there remain significant technical difficulties and fundamental challenges in synthesizing ordered nanostructured materials using these approaches.

Nanocasting synthesis using mesoporous silica as a hard template has attracted tremendous interest as a platform for developing efficient electrocatalysts based on earth-abundant metals [21]. The hard templating route has been successfully developed to prepare high-surface-area mesoporous multi-metal materials for OER from mixed oxides [22], perovskites [23], and phosphides [24]. Various synthesis routes for ordered mesoporous transition metal nitrides by nanocasting have also been developed [25]. This generally takes one of two synthetic routes: direct nitridation of mesoporous oxides or the transformation of mesostructured metal oxides/silica composites to nitrides/silica composites. Though there has been some success with mixed metal materials, to the best of our knowledge, mixed metal nitrides have not been significantly developed. By combining the benefits of nanocasting, with the synergetic effects of the electronic structure derived from multi-metal nitrides, one can achieve highly efficient mesoporous OER electrodes from a facile approach to achieve high catalyst loading.

Herein, we developed a facile two-step nanocasting–nitridation strategy to achieve a bimetal nitride Ni_3_FeN with ordered mesoporous structures, uniform mesopores, large pore volume, and large surface areas. Benefiting from both high catalyst loading and large contact area between the catalyst and electrolytes, this mesoporous Ni_3_FeN catalyst exhibits highly efficient and stable catalytic activity toward the OER in an alkaline solution. It exhibits a very low overpotential (259 mV) to achieve a 10-mA cm^−2^ geometric current density, which is lower than those of IrO_2_, RuO_2_, and an ordered mesoporous Ni_3_N electrocatalyst.

## Experimental

### Synthesis of Mesoporous Oxides

Ordered mesoporous NiO and Ni_3_FeO_*x*_ were synthesized following a nanocasting route with KIT-6 as the hard template according to the procedure reported by Deng et al. [26]. Typically, 0.5 g of KIT-6 and 0.8 M of metal nitrates as a precursor (Ni(NO_3_)_2_·6H_2_O, Fe(NO_3_)_3_·9H_2_O) were dispersed in 3.6 mL of ethanol in a weight ratio of 1:0 and 3:1 for NiO and Ni_3_FeO_*x*_ oxides, respectively. After 30 min of stirring at room temperature, ethanol was removed by evaporation through heating of the mixture overnight at 60 °C. Afterward, the resulting precursor ion filled mesoporous silica composite was heated in a ceramic crucible in an oven at 250 °C for 4 h to completely decompose the nitrate species. The impregnation step with the metal salt solution was repeated in order to achieve higher loadings. After evaporation of the solvent, the resulting metal precursor/silica composites were calcined at 550 °C for 6 h. The silica template was removed by treatment with 2 M NaOH solution, washed with deionized water and ethanol, and then dried at 60 °C for 12 h.

### Synthesis of Mesoporous Ni_3_N

The as-obtained mesoporous NiO (100 mg) was heated at 370 °C with a heating rate of 4 °C min^−1^ under a flowing pure ammonia atmosphere (1 bar, 200 sccm) and maintained for 4 h in a tube furnace. The furnace was then cooled down naturally to room temperature in a flowing ammonia environment.

### Synthesis of Mesoporous Ni_3_FeN

The nickel–iron nitride was obtained from the mesoporous Ni_3_FeO_*x*_ mixed oxides precursor via a similar nitridation reaction: 200 mg of the porous Ni_3_FeO_*x*_ precursor was heated with a heating rate of 4 °C min^−1^, maintained at 400 °C for 4 h under a flowing NH_3_ atmosphere, and then allowed to cool to room temperature.

### Zn–Air Battery (ZAB) Measurements

The Zn–air battery (ZAB) tests were performed with a homemade Zn–air cell. The air cathode was made by spraying catalyst ink onto carbon paper with a gas diffusion layer, with a catalyst loading of 0.25 mg cm^−2^. The catalyst ink was prepared by ultrasonically dispersing a mixture of 6 mg Ni_3_FeN, 6 mg iron phthalocyanine (FePc), 12 mL ethanol, and 100 μL of 5 wt% Nafion solution. For comparison, a mixture of 40% Pt/C and RuO_2_ (mass ratio 1:1) with the same catalyst loading on carbon paper was used as the catalytic layer. A polished Zn foil (thickness 0.3 mm) was used as the anode, and the electrolyte was 6.0 M KOH containing 0.20 M Zn(Ac)_2_. A Land CT2001A system was used to carry out the cycling test with a 10-min rest time between each discharge/charge cycle at a current density of 10 mA cm^−2^. Each discharge/charge period was set to be 30 min. The charge and discharge polarization curves were carried out using the PINE electrochemical workstation (Pine Research Instrumentation, USA).

Further details regarding the characterization of the catalysts are available in the Supporting Information.

## Results and Discussion

### Characterization of Mesoporous Electrocatalysts

**Fig. 1:**
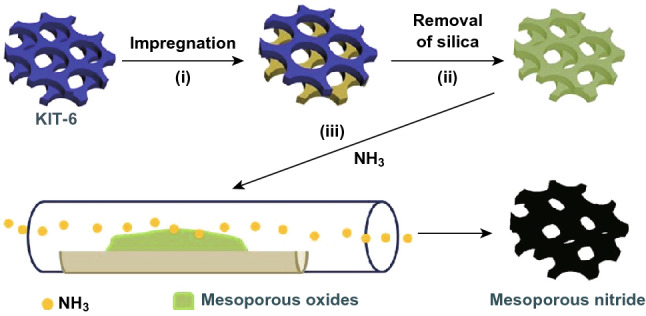
Schematic illustration of the synthesis strategy for ordered mesoporous bimetal Ni_3_FeN through a nanocasting route

As shown in Fig. 1, highly ordered mesoporous oxides were synthesized through a nanocasting route from mesoporous silica using 3D cubic KIT-6 silica as templates [27]. In the second step, after nanocasting, the silica template is removed, leaving behind a 3D mesoporous metal oxide replica. Then, the mesoporous replica was subsequently nitridized under an ammonia atmosphere.

**Fig. 2:**
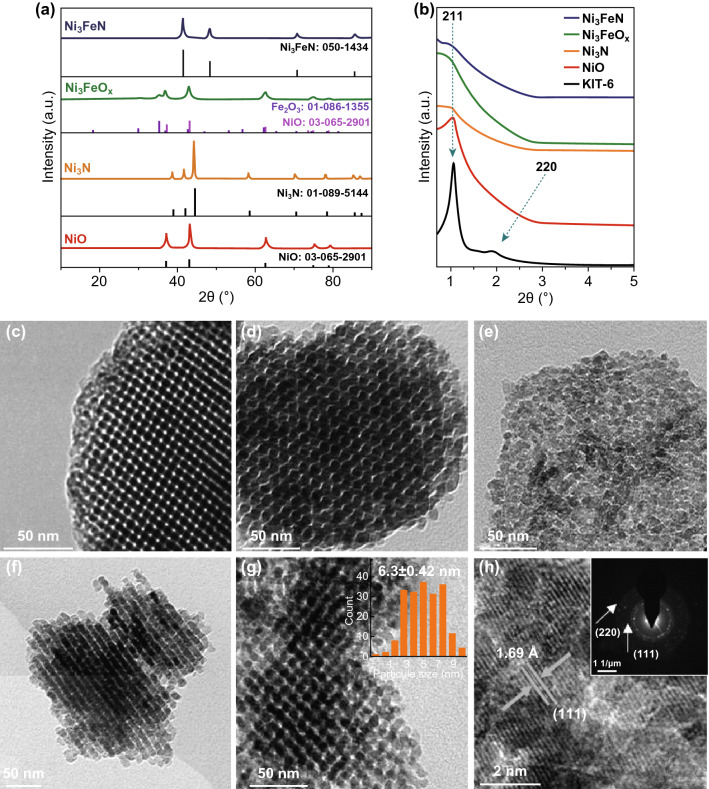
**a** Wide-angle XRD patterns of ordered mesoporous NiO, Ni_3_N, Ni_3_FeO_*x*_ mixed oxide, and Ni_3_FeN and their standard XRD JCPDS data. **b** Low-angle powder XRD patterns of KIT-6 and mesoporous materials replicated from KIT-6 silica. TEM images of **c** KIT-6 silica template along the [111] direction, **d** NiO, **e** Ni_3_FeO_x_, **f** Ni_3_N, and **g** Ni_3_FeN (inset: the corresponding particles sizes distributions); **h** HRTEM of Ni_3_FeN (inset: the corresponding FFT)

To verify the formation of crystalline phases, the mesoporous oxides replica and their corresponding nitrides were investigated by wide-angle X-ray diffraction (XRD). As shown in Fig. 2a, after flowing ammonia over the mesoporous NiO, the diffractogram shows completely different diffraction peaks, which are consistent with hexagonal Ni_3_N (JCPDS Card No. 01-089-5144) [28]. As well for the Ni_3_FeO_*x*_ precursor, annealing at 400 °C in ammonia results in a complete conversion to an iron-nickel nitride mesostructure. The resulting XRD pattern shows only the diffraction peaks that are indexed to Ni_3_FeN (JCPDS Card 50-1434). The diffraction peaks correspond to (111), (200), (220), and (311) planes of face-centered cubic (fcc) Ni_3_FeN [29, 30]. None of the samples show the characteristic peak at 23° from amorphous silica (Fig. S1), confirming the complete removal of the silica template. As shown in Fig. 2b, the low-angle diffractogram of the calcined KIT-6 shows two diffraction peaks which correspond, respectively, to the (211) and (220) reflections of a 3D Ia3d cubic structure [31, 32]. Compared with the template, all diffractograms of mesoporous oxide intermediates and their corresponding nitrides show a similar diffraction peak assigned to the (211) reflection, indicating that the ordered mesostructured is preserved. Transmission electron microscopy (TEM) analysis reveals the morphology of the pristine KIT-6 template and different as-synthesized mesoporous replicas and nitrides, as shown in Fig. 2c. The KIT-6 template consists of a highly ordered and interconnected mesopore system with a pore size diameter in the 5–6 nm range, resulting in the sharp diffraction features observed in Fig. 2b. This is also supported by the BET analysis (Fig. S2). After nanocasting, it can be observed that NiO (Fig. 2d) and Ni_3_FeO_*x*_ (Fig. 2e) exhibit an ordered framework and uniform nanoparticles with a narrow size range ~ 7 nm. Figure 2f shows the morphology of the mesoporous Ni_3_N. We can still clearly see that the ordered mesostructure and uniform particles of the mother material, NiO, were maintained after conversion to nitride. The diameters of those particles are approximately 6.9 ± 0.4 nm (Fig. S3f), which is in good agreement with the mesopore diameter of the KIT-6 (5–10 nm). This indicates that the transformation of NiO into Ni_3_N maintained the confined structure of the pore channels [33]. For the as-synthesized mesoporous bimetallic Ni–Fe nitride (Fig. 2g), the TEM micrograph shows that the Ni_3_FeN partially consists of large domains with an ordered framework and uniform nanoparticles with an average size 6.3 ± 0.4 nm arranged uniformly throughout the material as expected (inset of Fig. 2g). Nitrogen sorption isotherms of Ni_3_N and Ni_3_FeN (Fig. S4) indicate that both of them display type IV sorption isotherm and exhibit hysteresis loops, as is typical for mesoporous materials. The template-free mesostructured nitrides have BET surface area of 50 and 52 m^2^ g^−1^ with pore size distributions ranging from 5 to 8 nm, for Ni_3_N and Ni_3_FeN, respectively.

Furthermore, the high-resolution TEM (HRTEM) image of Ni_3_FeN (Fig. 2h) indicates that the fabricated mesoporous ternary nitride is well crystallized, and the integrated crystal lattice pattern suggests that a single crystal exists in this domain. The *d*-spacing of the plane is 1.69 Å, corresponding to the (111) plane of Ni_3_FeN [34]. Typical selected area electron diffraction (SAED) pattern (inset of Fig. 2h) displays highly resolved concentric rings and regular spots, suggesting the high crystallinity of the sample in agreement with the HRTEM results. In addition, the energy-dispersive spectrum (EDS) elemental mapping (Fig. S7b) reveals a uniform distribution of the Ni, Fe, and N atoms in the selected field (similar to the mother oxide as shown in Fig. S6). The molar ratio of Ni/Fe in Ni_3_FeN, determined from the EDX spectrum (Fig. S5c), is around 3.012: 0.988, close to the expected Ni/Fe ratio of 3:1.

**Fig. 3:**
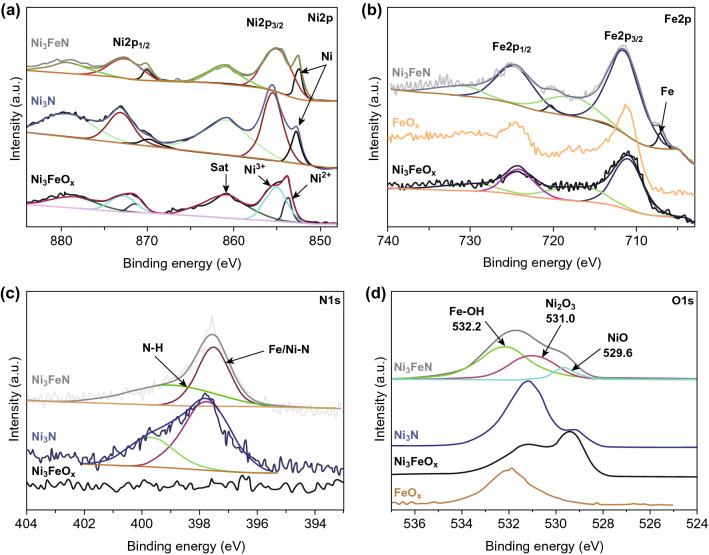
XPS high-resolution scan of **a** Ni 2*p*, **b** Fe 2*p*, **c** N 1*s*, and **d** O 1*s* for various phases

X-ray photoelectron spectroscopy (XPS) was employed to characterize the chemical valence states of the various elements of the bimetallic nitride catalyst. The XPS survey spectra for Ni_3_FeN samples (Fig. S8) confirm the presence of Ni, Fe, and N, with O and C peaks resulting from surface oxidation [35]. The Ni 2*p* high-resolution scans show the expected doublet Ni 2*p*_1/2_ and Ni 2*p*_3/2_ for various samples (Fig. 3a). As can be seen, the nitrated mesoporous materials, Ni_3_N and Ni_3_FeN, show a low binding energy peak centered at 852.6 eV in Ni 2*p*_3/2_ not visible in the mother oxide template. This peak can be attributed to the metallic state of Ni [36], which results from the formation of nitride [8, 28, 37]. Additionally, there are two broad peaks centered at 855.3 and 861.2 eV in Ni 2*p*_3/2_ with corresponding 872.7 and 879.9 eV peaks in Ni 2*p*_1/2_. These can be described by oxidized Ni species and Ni satellite peaks, respectively [36]. Given the diffraction results, which had no significant oxide phases, this suggests that the Fe–Ni ternary nitrides samples are partially oxidized at the surface [8, 38]. The amount of metallic Ni appears to be increased with the addition of iron, as shown by the relative intensity of the metallic peak to the oxidized peaks. For Ni_3_FeO_*x*_, it is clear that the Ni oxide can be described by two components, corresponding to predominately Ni^2+^ (NiO) and predominantly Ni^3+^ (Ni_2_O_3_) oxidation states [39], while the nitrides only have one dominate oxide phase. The nitridation process seems to preferentially affect the Ni^2+^ phase, which disappears with nitridation, while the Ni^3+^ phase persists. This behavior is also supported by the O 1*s* high-resolution scans (Fig. 3d), where the O 1*s* of Ni_3_FeO_*x*_ has a strong feature at 529.6 eV ascribed to NiO [39], which greatly diminished with nitridation. For the mixed metal nitride, there is also evidence of an iron hydroxide phase, at 532.2 eV [40, 41]. Figure 3d shows a pure FeO_*x*_ phase consisting of γ-Fe_2_O_3_ nanoparticles as a reference, showing a similar surface hydroxide phase [41]. Zhu et al. [38] also observed this tendency of the addition of Fe to shift the oxidation state of mixed metal oxides, which is dominated by M–OH bonds.

Figure 3b displays high-resolution Fe 2*p* spectra of Ni_3_FeN, Ni_3_FeO_*x*_, as well as a reference γ-Fe_2_O_3_ spectra. Again, the nitridation results in the formation of a new peak at 706.9 eV, which can be assigned to metallic iron nitride [35]. However, in this case the signal is dominated by the oxide related and satellite peaks at 711.6 and 717.9 eV [35, 42]. These features are consistent with γ-Fe_2_O_3_, though it is difficult to unambiguously assign the oxidation states in iron oxides [41–43]. In the N 1*s* region (Fig. 3c), the spectrum can be deconvoluted into a combination of two components at 397.5 and 399.2 eV for nitrided samples [44].

### Electrocatalytic Oxygen Evolution Reaction

**Fig. 4:**
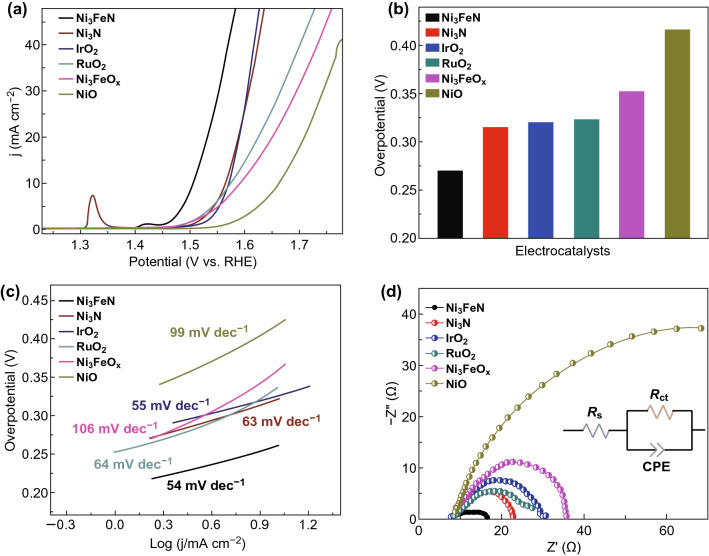
**a** OER polarization curves in O_2_-saturated 1 M KOH (sweep rate: 5 mV s^−1^; rotation speed: 1600 rpm); **b** OER overpotentials at a current density of 10 mA cm^–2^, **c** Tafel plots, **d** Nyquist plots obtained at 270 mV overpotential on NiO, Ni_3_Fe mixed oxide, IrO_2_, RuO_2_, Ni_3_N, and Ni_3_FeN (inset shows the equivalent circuit)

An efficient oxygen evolution catalyst with superior catalytic activity requires an early-onset potential with high kinetic current density, small Tafel slope, and good durability [45]. To identify whether mesoporous iron–nickel nitride is a promising electrocatalyst for OER, electrochemical measurements were carried out by linear sweep voltammetry (LSV) on all the synthesized electrocatalysts and benchmark catalysts with the same loading density of ~ 0.203 mg cm^−2^. The upper bound of the potential was set at 1.8 V versus RHE (reversible hydrogen electrode) to avoid the detachment of the catalytic layer and interference with the measurement as a result of excessive oxygen evolution. As displayed in Fig. 4a, OER activity curves of the as-synthesized transition metal nitrides catalysts, transition metal-based oxides, and commercial RuO_2_ and IrO_2_ were all studied under the same conditions for direct comparison.

The as-prepared mesoporous bimetallic electrocatalyst iron–nickel nitride exhibited an early-onset potential of ~ 1.45 V versus RHE, high kinetic current density at a fixed potential, and the lowest overpotential (259 mV) (Fig. 4b) to achieve a current density of 10 mA cm^−2^. Compared to the nickel iron mixed oxides (352 mV), IrO_2_ (320 mV), RuO_2_ (322 mV), and NiO (415 mV), the nitrides showed the lowest overpotentials, demonstrating that the conversion of oxides to mesoporous nitrides can significantly improve the catalytic performance. Moreover, the mesoporous binary Ni_3_N exhibited slightly inferior electrocatalytic activity with an overpotential of 315 mV. As predicted, after addition of Fe to form a ternary compound, the catalytic activities of metal nitrides were enhanced. The outstanding electrocatalytic performance likely originates from: (1) the synergetic effect between bimetal atoms, as reported in other bimetallic alloys such as Fe_x_Ni_1−*x*_OOH [46], Ni_0.51_Co_0.49_P [9], Ni–MnO/rGO aerogel [47], and NiCo_2_S_4_ [7]; (2) hierarchical porosity composed of mesopores connected with macropores (or larger mesopores) facilitating fast mass transport, resulting in improved electrode performance [48–52]; or (3) a combination of these factors.

It is important to note that in the polarization curve of Ni_3_N, an apparent oxidation peak centered at about 1.32 V can be observed prior to the onset of oxygen evolution. This could be assigned to the oxidation redox peaks of Ni^2+^/Ni^3+^ conversion during the OER in alkaline electrolytes, which have been investigated in detail in most of Ni-based electrocatalysts [53, 54]. The shift to around 1.4 V and decrease in total Ni oxidation peak intensity in the LSV curve of Ni_3_FeN are likely attributed to the incorporated Fe suppressing the transformation of Ni(OH)_2_ to NiOOH [55], which is consistent with other bimetallic alloys (Ni,Co)S_2_ [56] and Fe–Ni hydroxide [55]. This suppression is also supported by the observed preferential surface oxidation of Fe in the XPS results. Additionally, it is likely that the nitridation process itself contributes to this effect, as it was mainly the Ni^2+^ that was converted into nickel nitride. With a limited abundance of Ni^2+^, there is a lower content of Ni(OH)_2_ in ternary nitride. These two effects likely result in a reduction in the number of active sites available for nickel oxidation. Louie et al. [57] also observed the decrease in average intensity for the oxidation state of Ni in NiOOH with the incorporation of Fe in Ni–Fe films.

The Tafel slope and current densities exchange were determined to evaluate the kinetic process. As shown in Fig. 4c, the Tafel plots derived from polarization curves demonstrate that the Ni_3_FeN exhibits the smallest Tafel slope of 54 mV dec^−1^ among these tested catalysts, which indicates efficient electron transfer and rapid kinetic activities. This is similar to the commercial heavy metal catalyst, but substantially smaller than mesoporous NiO and nickel–iron mixed oxides (99 and 106 mV dec^−1^, respectively). The ternary Ni_3_FeN also has a lower Tafel slope than the binary Ni_3_N (63 mV dec^−1^), demonstrating more rapid kinetic activity, supporting the improved catalytic performance.

**Fig. 5:**
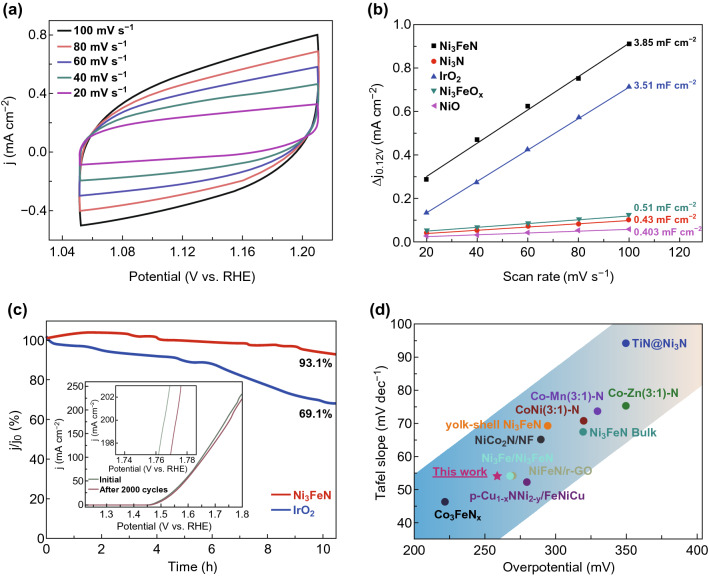
**a** Cyclic voltammograms for Ni_3_FeN with a scan rate of 20, 40, 60, 80, and 100 mV s^−1^ in 1 M KOH. **b** The capacitive current density at 0.12 V as a function of the scan rate for NiO, Ni_3_N, IrO_2_, Ni_3_FeO_*x*_ and Ni_3_FeN electrocatalysts. **c** Chronopotentiometry of Ni_3_FeN and IrO_2_ at an overpotential of 370 mV (inset shows polarization curves recorded for the catalysts before and after 2000 cycles of CV scans. **d** Comparison of the electrocatalytic performance (Tafel slopes and the overpotentials reaching a current density of 10 mA cm^−2^) of mesoporous Ni_3_FeN with other nitrides reported recently

To investigate the reaction kinetics occurring at the electrode–electrolyte interface during the OER, electrochemical impedance spectroscopy (EIS) was also measured in a three-electrode system in 1.0 M KOH. On basis of the Nyquist plots (Fig. 4d), the smallest semicircle for Ni–Fe nitride suggests the lowest charge-transfer resistance for OER at an overpotential of 1.5 V compared with the other mesoporous catalysts, which is consistent with the superior OER activity and smaller Tafel slope. Double-layer capacitance (*C*_dl_) measurements were performed to evaluate the electrochemically active surface area (ECSA) of the mesoporous Ni_3_FeN catalyst through electrical double-layer capacitance (EDLC) measurements in 1 M KOH electrolyte [56, 58, 59]. The charging currents were collected at different scan rates (20, 40, 60, 80, and 100 mV s^–1^) as shown in Fig. 5a.

The mesoporous Ni_3_FeN catalyst provided high cathodic (*i*_c_) and anodic (*i*_a_) current densities at each rate scan, consistent with a much greater active surface area than the other mesoporous catalysts (Fig. S9). The double-layer capacitance value of Ni_3_FeN was estimated around 3.85 mF cm^–2^ calculated from the linear plots between the scan rate and current density at 0.12 V (Fig. 5b). Ni_3_FeN has significantly higher capacitance, reflecting a high electrochemical surface and, consequently, a high surface roughness, compared to Ni_3_N (0.43 mF cm^−2^), NiO (0.401 mF cm^−2^), and Ni_3_FeO_*x*_ (0.51 mF cm^−2^) (Fig. S9). This is also higher than the commercially available IrO_2_ (3.51 mF cm^−2^). The larger ECSA should result in better exposure and enhanced utilization of the catalytic active sites, thus giving rise to the observed improved OER activity [60].

Stability is another important consideration for an efficient OER electrocatalyst in alkaline solution. The long-term stability of the Ni_3_FeN and commercially available IrO_2_ measured at a fixed overpotential of 370 mV during continuous operation for 10 h is summarized in Fig. 5c. Current density values negligibly declined for Ni_3_FeN (with a retention rate of 93.1%) compared to IrO_2_ (69.1%) over the same time period. In addition, as displayed in the inset of Fig. 5c, Ni_3_FeN has stable OER catalytic performance with negligible degradation for the OER process and onset potential after continuous scanning for 2000 cyclic voltammetry cycles. At a current density of 200 mA cm^−2^, the overpotential decreased by only 7 mV over that time. Figure 5d compares the OER activity and OER kinetics estimated by Tafel plots with other mixed metal nitride catalysts. Our catalyst reveals a low overpotential along with a small Tafel slope, which is superior to most of the recently reported transition metal nitrides for the OER. The superior OER activity and good stability of the as-prepared Ni_3_FeN likely result from a combination of the following factors: (1) highly active electrocatalyst contact area can provide abundant accessible active sites, (2) the hierarchical porosity facilitates fast mass transport [61, 62], (3) the presence of nitrides facilitates electron/proton transfer, as well as smooth ion diffusion and transportation [63], (4) the synergistic effects of mixed metals in the ternary catalyst adjust the electronic structure and improve precipitation energies compared to single-metal materials, and (5) the favorable in situ oxidation of both metals species may play an important role in improving the catalytic activity, with the hydroxide species offering favorable active sites for hydroxyl adsorption [64]. Compared to other catalysts, particularly Co_3_FeN_*x*_, Ni_3_FeN combines excellent OER activity, and a highly crystalline and mesoporous structure with monophase formation, with a facile synthesis method for low toxicity iron (compared to cobalt), all of which make our electrocatalyst a promising alternative for green energy hydrogen production.

### Rechargeable Zn–Air Battery

**Fig. 6:**
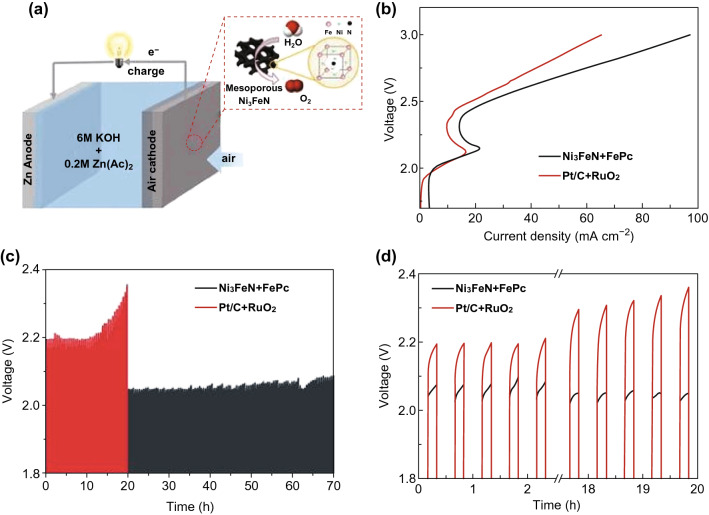
**a** Schematic illustration of a rechargeable Zn-air battery (ZAB) using FePc and mesoporous Ni_3_FeN as ORR and OER electrocatalysts in the air cathode, respectively (FePc not shown here); **b** charge polarization curves for a battery with Ni_3_FeN + FePc and Pt/C + RuO_2_ as the air electrode catalysts; **c**, **d** charge/discharge cycling of a rechargeable ZAB based on Ni_3_FeN + FePc and Pt/C + RuO_2_ at 10 mA cm^−2^. Only the charge curves were shown here to highlight the advantages of mesoporous Ni_3_FeN used as OER electrocatalysts in a rechargeable ZAB

To investigate the practical performance of mesoporous Ni_3_FeN as OER electrocatalyst, a rechargeable Zn–air battery (ZAB) was made by mixing iron phthalocyanine (FePc) and Ni_3_FeN with a mass ratio of 1:1 as the air cathode (Fig. 6a). The RDE measurements confirmed that FePc shows a high intrinsic ORR activity (Fig. S10), superior to commercial Pt/C in 0.1 M KOH [65, 66].

Figure 6b displays the charge polarization curves of the two air electrodes (FePc + Ni_3_FeN and Pt/C + RuO_2_) in ZAB, implying a better charge performance of Ni_3_FeN than RuO_2_ as confirmed by its higher current densities at the same potential. In addition, the charge–discharge cycling tests were carried out at current density of 10 mA cm^−2^. As shown in Fig. 6c, d, Ni_3_FeN + FePc exhibits a long cycle life as evidenced by stable fixed response even after 70 h, which is much longer than that (~ 20 h) of the mixed Pt/C + RuO_2_ battery. More importantly, the charging voltage of Ni_3_FeN is always lower than that of RuO_2_, even decaying slightly over time. After the charge and discharge cycle was performed for 20 h, the charging voltage of Ni_3_FeN is only 2.05 V, much smaller than 2.36 V of RuO_2_. This suggests the potential application of mesoporous Ni_3_FeN as an OER electrocatalyst superior to the conventional electrodes in a rechargeable ZAB.

## Conclusions

This work conclusively shows the synthesis of three-dimensional (3D) ordered mesoporous Ni_3_N and Ni_3_FeN ternary nitrides from intermediate mesostructured metal oxide replicas with high surface areas through a hard templating method and subsequent nitridation. The products of the facile two-step synthesis process possessed a high degree of crystallinity, large specific areas, and uniform mesopore sizes (~ 6 nm). Ternary Ni_3_FeN shows outstanding OER performance with very low overpotential (259 mV) to achieve a 10-mA cm^−2^ geometric current density with small Tafel slope and considerable durability toward OER, which is superior to both mesoporous Ni_3_N electrocatalysts and commercially available IrO_2_/RuO_2_. The enhanced catalytic performance of ordered mesoporous Ni_3_FeN likely derives from its intrinsic activity, hierarchical porosity, abundant active sites, large contact area between the catalyst and electrolytes, synergistic tuning of electronic structure and charge transport characteristics, and favorable oxidation behavior. Furthermore, when combining FePc and Ni_3_FeN as the air cathode of a rechargeable ZAB, mesoporous Ni_3_FeN shows a much lower charge voltage and a longer charge life than RuO_2_ in a ZAB. To summarize, these findings represent an attractive and efficient pathway toward the development of high-surface-area catalysts for electrical–chemical energy conversion. Such a platform also extends the fundamental understanding of the structure–property relationships of metal nitrides. It paves the way for the synthesis of other mesoporous ternary nitrides, which could be used in various applications, such as rechargeable ZABs and supercapacitors. Such a facile approach has the potential to provide the low-cost alternative energy sources necessary for the next-generation green technologies.

## Electronic supplementary material

Below is the link to the electronic supplementary material.

Supplementary material 1 (PDF 940 kb)
