# Supplementary material for: Mesoporous Ternary Nitrides of Earth-Abundant Metals as Oxygen Evolution Electrocatalyst
Source: Nanomicro Lett. 2020 Mar 26;12:79. doi: 10.1007/s40820-020-0412-8 (PMC7770804; doi:10.1007/s40820-020-0412-8)
Supplement: Supplementary file 1 — Supplementary material 1 (PDF 940 kb) [file 40820_2020_412_MOESM1_ESM.pdf]

Supporting Information for

Mesoporous Ternary Nitrides of Earth Abundant Metals as Oxygen Evolution Electrocatalyst

Ali Saad[1], Hangjia Shen[1], Zhixing Cheng[1], Ramis Arbi[3], Beibei Guo[4], Lok Shu Hui[3], Kunyu Liang[3], Siqi Liu[1], John Paul Attfield[5], Ayse Turak[3,]*, Jiacheng Wang[2, 4,]*, Minghui Yang[1, 2,]*

[1]Ningbo Institute of Materials Technology and Engineering Chinese Academy of Sciences Ningbo 315201, Zhejiang Province, People's Republic of China

[2]Center of Materials Science and Optoelectronics Engineering, University of Chinese Academy of Sciences, Beijing 100049, People's Republic of China

[3]Department of Engineering Physics, McMaster University, Hamilton, L8S 4L7, Canada

[4]State Key Laboratory of High Performance Ceramics and Superfine Microstructure Shanghai Institute of Ceramics, Chinese Academy of Sciences1295 Dingxi Road, Shanghai 200050, People's Republic of China

[5]Centre for Science at Extreme Conditions and EaStCHEM School of Chemistry, University of Edinburgh, Kings Buildings, West Mains Road, Edinburgh EH9 3JJ, UK

*Corresponding authors. E-mail: turaka@mcmaster.ca (Ayse Turak); jiacheng.wang@mail.sic.ac.cn (Jiacheng Wang); myang@nimte.ac.cn (Minghui Yang)

S1 Materials

Poly(ethylene oxide)-b-poly(propylene oxide)-b-poly(ethyleneoxide) [$EO_{20}PO_{70}EO_{20}$, Pluronic P123, Mw =5800], tetraethylorthosilicate (TEOS, 98%), n-Butanol, concentrated hydrochloric acid (37% wt%, AR) were purchased from Aladdin Industrial Corporation (Shanghai, China), sodium hydroxide (NaOH 90%), Iron (III) nitrate nonahydrate Fe(NO3)39H2O, Nickel (II) nitrate hexahydrate $Ni(NO_3)_2$ $6H_2O$, potassium hydroxide (KOH, 99%) , RuO_2 and IrO_2 (20% Iron Vulcan XC-72) and Nafion were purchased from Alfa Aesar. All chemicals were used as received without any further purification. Deionized water and ethanol are used throughout the experiment.

S2 Synthesis of KIT-6 Mesoporous Silica

The mesoporous KIT-6 template was synthesized according to the procedure of Rossinyol and co-workers [S1]. First, 6 g of nonionic triblock copolymer Pluronic P123 was dissolved in a mixture of 220 ml of water and 12 g of HCl (37%). The solution was vigorously stirred for 6 h at 35 °C. After complete dissolution of Pluronic P123, 6 g of butanol was added to the acidic solution under continuous stirring for another hour. Next, 12.48 g of TEOS was rapidly added to the solution under continuous stirring for 24 h. Subsequently, the mixture was heated at 100 °C under static conditions for 24 h. The resultant white solids obtained after filtration was washed and dried for 12 h at 90 °C. Finally, the template was removed by calcination from as-synthesized KIT-6 at 550 °C and in the air for 6 h with a heating rate of 2°C min^{-1}.

S3 Structural Characterization

Powder X-ray diffraction (XRD) patterns were recorded on an Rigaku MiniFlex 600 using a powder X-ray diffractometer with Cu Kα radiation source (λ = 1.5418 Å, accelerating voltage 40 kV, and applied current 15 mA) (Japan) in a 2θ range from 10° to 90°. Low-angle X-ray

diffraction (XRD) data were recorded on Bruker AXS diffractometer System from 0.5 to 5 (2θ). Transmission electron microscopy (TEM) and high-resolution transmission electron microscopy (HRTEM) observations are conducted on a Jeol JEM-2100 (Japan) instrument equipped with a cold field emission gun and a Noran energy dispersive X-ray (EDX) unit at an acceleration voltage of 200 kV. For TEM measurements, samples were prepared by dispersion in ethanol with an ultrasonic bath. A few drops from these suspensions were gently deposited onto a carbon-covered copper grid, and the ethanol was allowed to evaporate. XPS measurements were recorded using an ESCALAB250 X-ray photoelectron spectrometer with a non-monochromatized Al-Kα X-ray as the excitation source, and the C1s component was set at 284.8 eV to calibrate the spectra. Surface area measurements are performed by nitrogen adsorption using Brunauere Emmet Teller (BET) area method whereas mesopore size was assessed by the Barrett−Joyner−Halenda (BJH) method from the desorption branch of the isotherm.

S4 Electrochemical Measurements

The electrochemical measurements were performed with on an MSR electrode rotator (Pine Instrument Co.) coupled with CHI 635A electrochemical workstation. Oxygen evolution reactions (OER) were performed in a three-electrode configuration with an Ag/AgCl (in a saturated KCl solution) electrode used as the reference electrode, platinum net as the counter electrode and a glassy carbon electrode coated with electrocatalysts as the working electrode. In this work, all electrochemical experiments were carried out at a scan rate of 5 mV s^{-1} at 25 ± 0.2 °C in 1.0 M KOH with the *iR* compensation (always 95%), with *R* about 7 Ω. The working electrodes were prepared first by dispersing 4 mg of catalysts in a mixed solution of 0.25 ethanol mL, 0.75 mL H_2O, and 25 μL 5 wt.% Nafion solution, followed by sonication for at least 30 min to form a homogeneous ink. Then, 10 μL of the resulting dispersion was dropped on the glassy carbon electrode (5 mm diameter, 0.196 cm^2 surface areas with an electrocatalyst loading of ~0.203 mg cm^{-2}). The measured potentials were converted to a reversible hydrogen electrode (RHE) according to the Nernst equation:

$$E_{RHE} = E_{Ag/AgCl} + 0.0591\, pH + 0.197$$

The overpotential (η) was calculated according to:

$$\eta(V) = E_{RHE} - 1.230$$

Cyclic voltammetry (CV) curves were obtained by a cycling scan at room temperature after purging with oxygen gas (99.9%) 30 min before each RDE experiment to ensure full oxygen saturation of the electrolyte. The working electrodes were scanned for several potential cycles until the signals were stabilized, and then the CV data were collected.

Prior to the impedance measurements, the electrodes were stressed in the same configuration under a potential of 1.55 V (vs. RHE) with frequency from 10 MHz to 100 mHz and amplitude of 5 mV. The Nyquist plots were thus obtained based on the electrochemical impedance spectroscopy data.

The double-layer capacitance, C_{dl}, of the electrodes – electrolyte interface was estimated by plotting $(j_a\text{-}j_c)/2$ vs. the scan rate [S2] such that:

$$C_{dl} = \frac{\Delta Q}{\Delta V} = \frac{j}{v}$$

All synthesized electrocatalysts and benchmark catalysts (IrO_2 and RuO_2) were evaluated in similar conditions by linear-sweep voltammetry (LSV) in 1.0 M KOH solution with the same catalyst loading of 0.2 mg cm^{-2} in the potential range from 1.1 to 1.8 V.

S5 Supplementary Figures

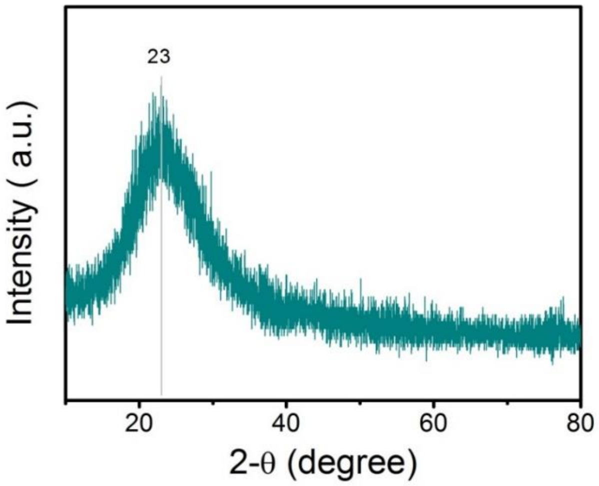

Fig. S1 Wide-angle powder XRD patterns of mesoporous KIT-6. One can note a very broad single peak at about 23° in 2theta with a Cu source, characteristic of amorphous silica. This peak disappears for all of the mesoporous oxides and their nitrides, confirming the successful removal of the template [S3].

To verify the formation of crystalline phases, the nanocast nickel monoxide sample XRD patterns as shown in **Fig.** 2a in the main manuscript display well-defined diffraction peaks which could all be indexed to NiO (JCPDS Card No. 03-065-2901). No other diffraction peaks can be observed, indicating a pure phase of NiO. Similarly, the pattern of mesoporous Ni_3FeO_x mixed-oxide displays main diffraction peaks at 30.27°, 35.69°, 37.31°, 43.34°, 57.40°, 63.01°, 74.54°, and 79.55°, which could be indexed to (206), (119), (226), (0012), (1115), (4012), (3315), and (4412) crystal planes of Fe_2O_3 phase (JCPDS card 00-025-1402), respectively. Among these main peaks, those at 37.26°, 43.29°, 62.88°, 75.42°, and 79.41° are coinciding with NiO crystal planes.

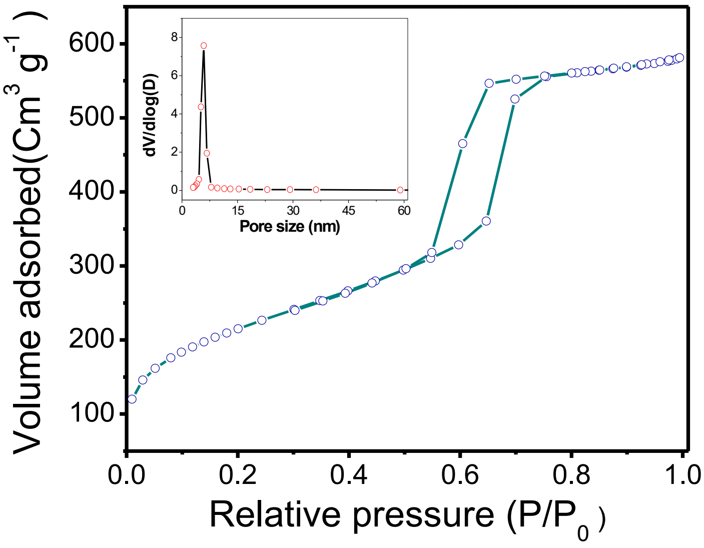

Fig. S2 Nitrogen adsorption/desorption isotherms of KIT-6 (the inset shows the BJH pore-size distribution). The specific surface (S_{BET}) values were obtained using the Brunauer–Emmett–Teller (BET) method [S4] while the mesopore size of the materials was determined by the Barrett–Joyner–Halenda (BJH) method [S5]. The synthesized KIT-6 template, have a large specific surface area of 768 $m^2 g^{-1}$ and a pore volume of 0.92 $cm^3 g^{-1}$. The BJH desorption average pore diameter is ca. 5.4 nm.

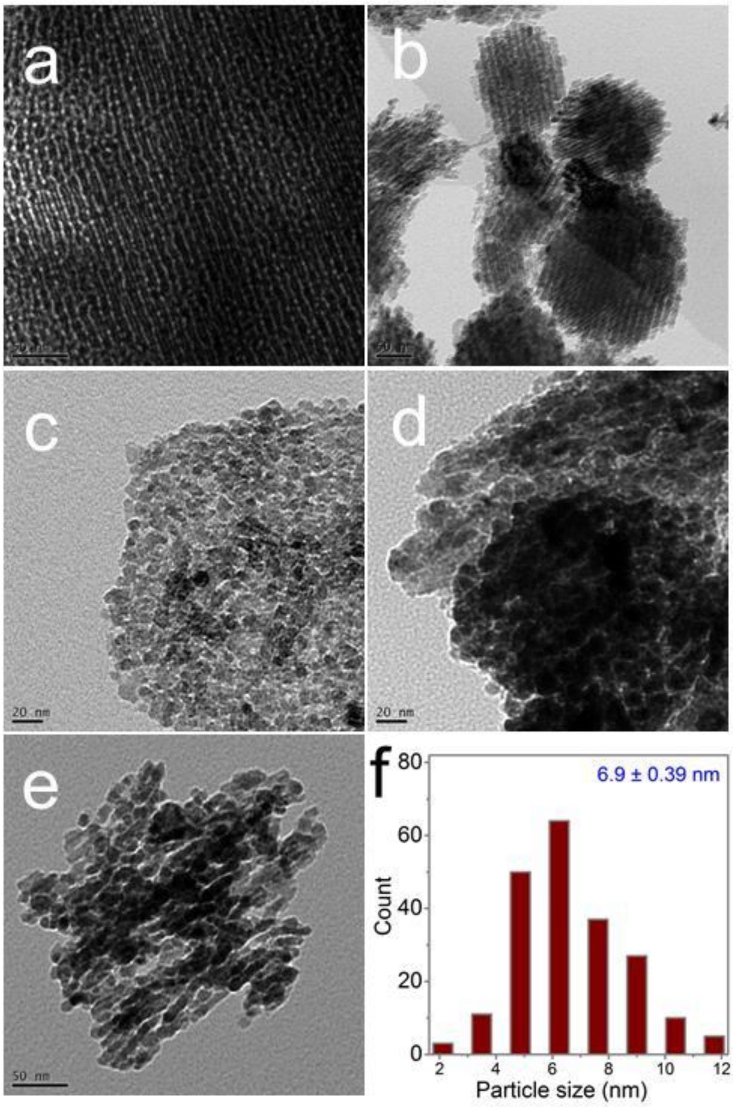

Fig. S3 TEM images of **a**) KIT-6, **b**) NiO, **c**) Ni_3FeOx, **d**) Ni_3N, **e**) and Ni_3FeN, **f**) particles sizes distributions for Ni_3N

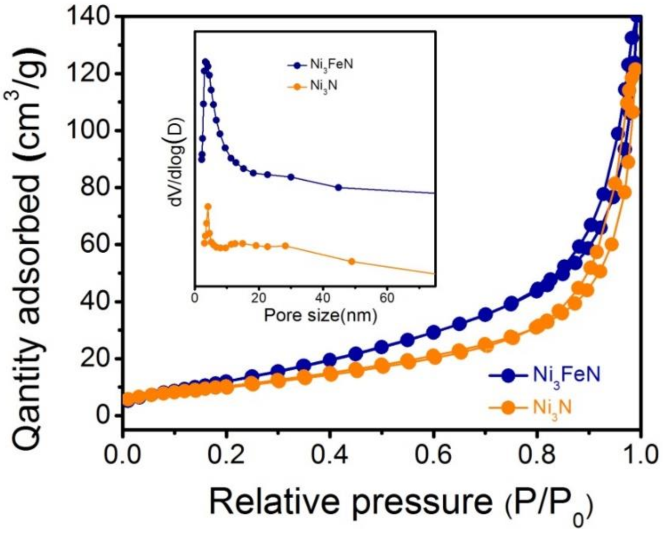

Fig. S4 Nitrogen adsorption/desorption isotherms at 77 K for Ni_3N and Ni_3FeN (pore size distribution inset) calculated with the adsorption branch

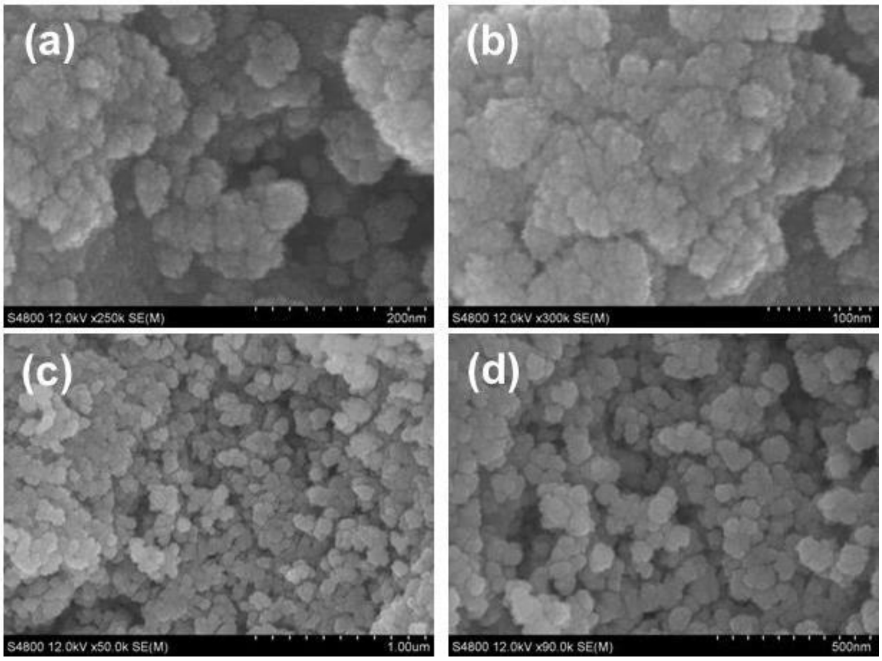

Fig. S5 SEM image of Ni_3FeOx precursor (**a, b**), Ni_3FeN (**c, d**). mixed Ni_3-Fe oxides and their corresponding bimetallic nitride consists of well-defined quasi-spherical NPs with an average size between 100 and 300 nm, matching well with the KIT-6 hard template morphology [S6].

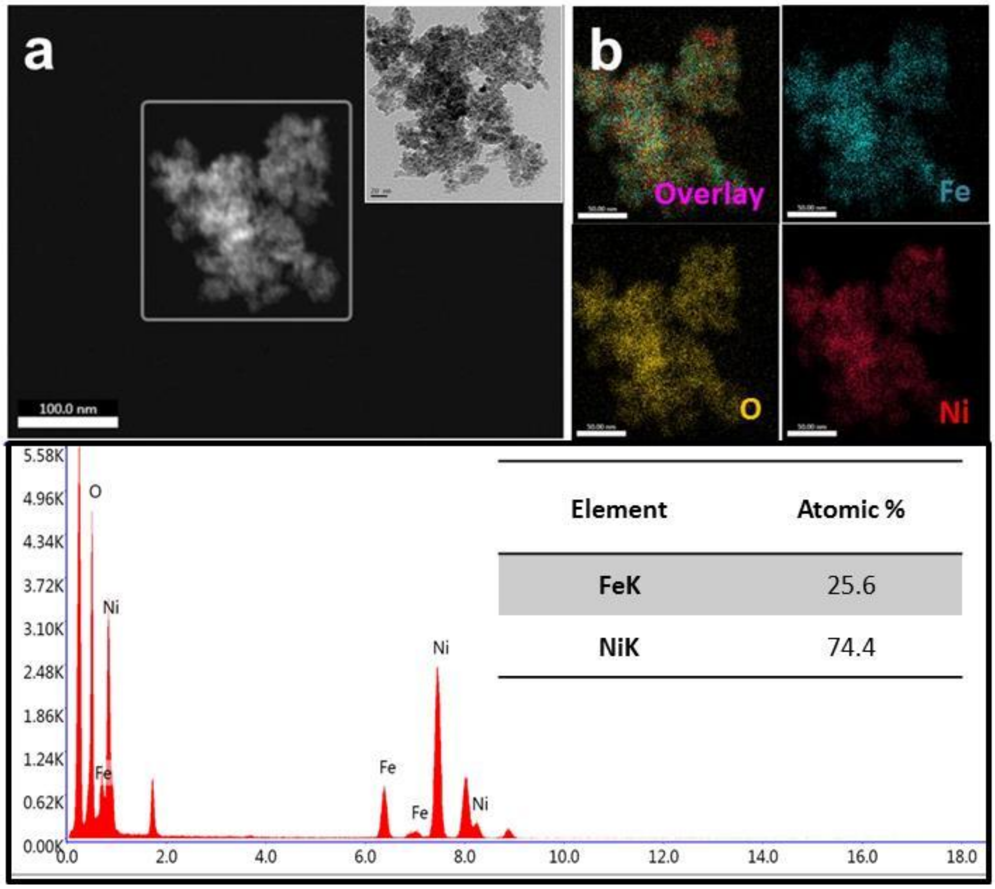

Fig. S6 EDS line scanning profiles of Ni_3FeO_x (**a**) Dark field image of scanned region (**b**) Elemental mapping images of Fe, O and Ni, and (**c**) EDX spectrum. EDS elemental mapping revealed a uniform distribution of the Ni, Fe, and O atoms in the selected field. The molar ratio of Ni/Fe in Ni_3FeO_x, determined from the EDX spectrum is around 2.97: 1.02, close to the expected Ni/Fe ratio of 3:1.

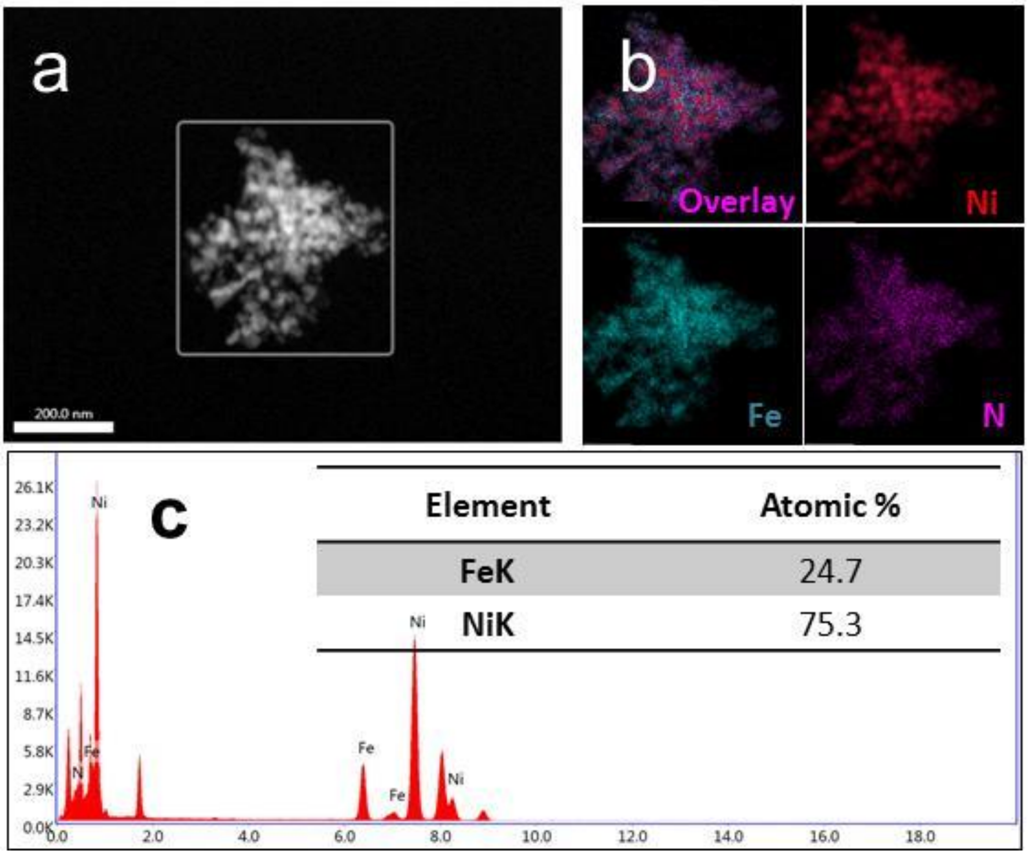

Fig. S7 EDS line scanning profiles of Ni_3FeN (**a**) Dark field image of scanned region (**b**) Elemental mapping images of Fe, Ni, and N, and (**c**) EDX spectrum

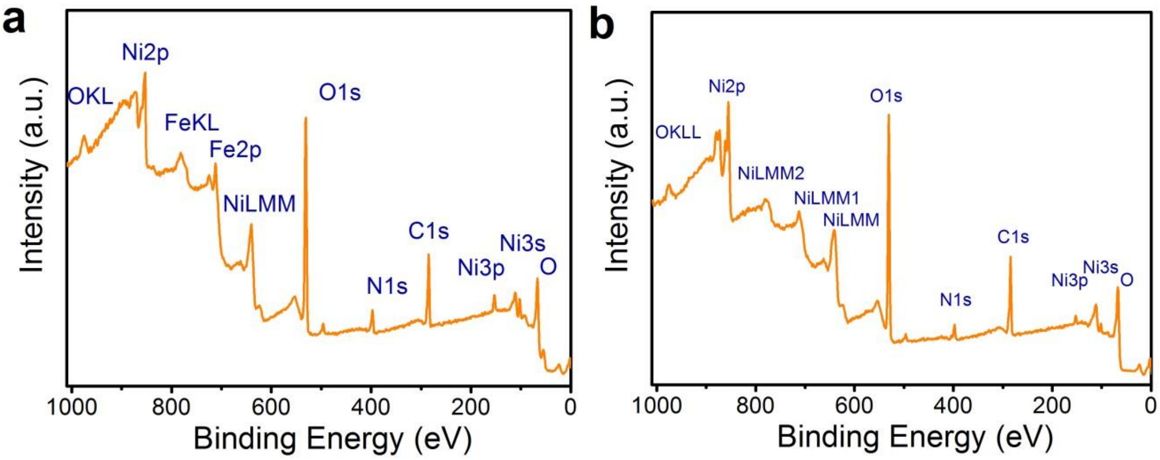

Fig. S8 XPS survey scan of Ni_3FeN (**a**) and Ni_3N (**b**)

In the N 1s region (**Fig.** 3c in the main document), the spectrum can be deconvoluted into a combination of two components at 397.5 and 399.2 eV for nitrided samples, while the mother oxide has no features in this region. The first component can be ascribed to the metal nitride; the small shoulder can be ascribed to NH moieties, probably resulting from the incomplete reaction of mesoporous oxides with NH_3 at the surface [S7].

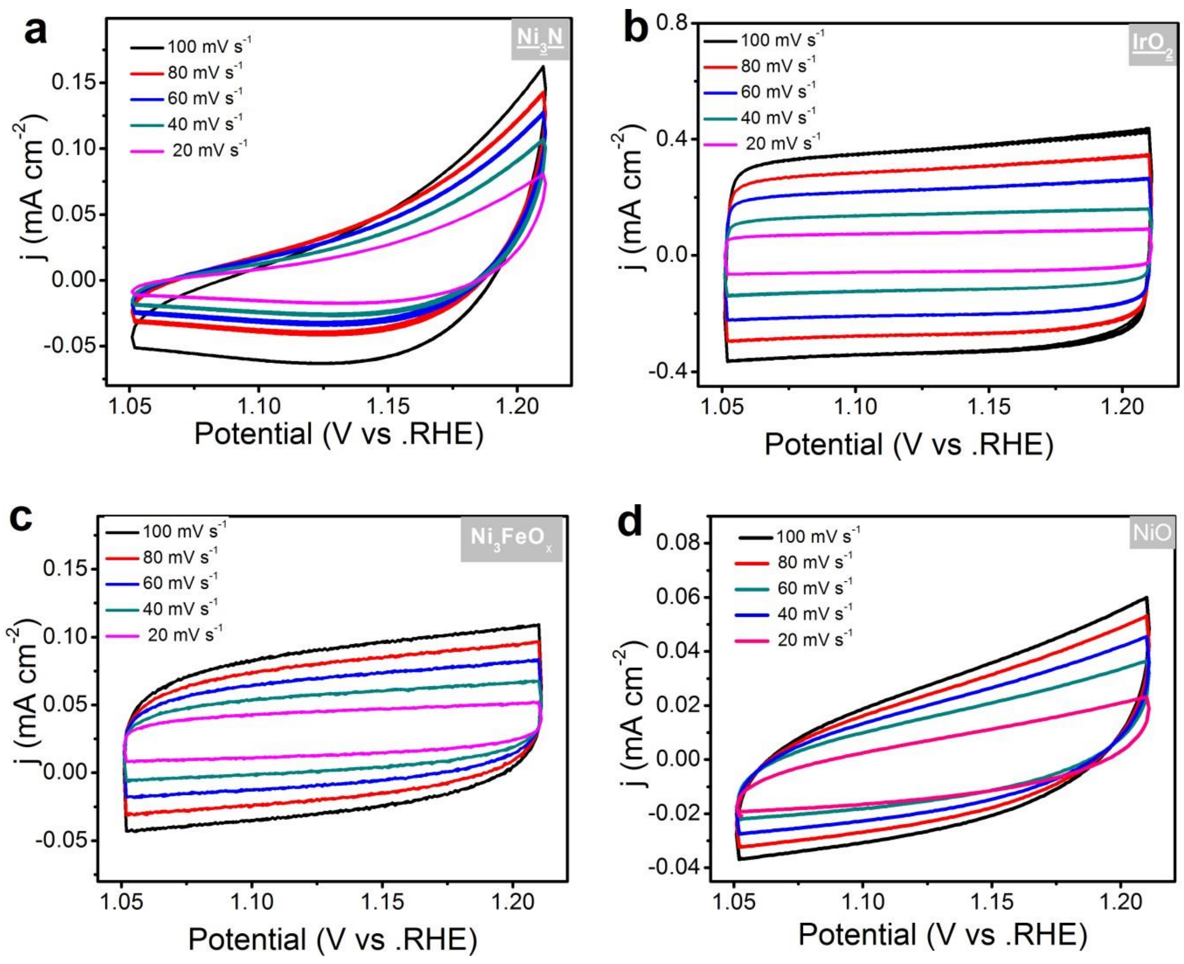

Fig. S9 Cyclic voltammograms with a scan rate of 20, 40, 60, 80, and 100 mV s^{-1} in 1 M KOH for Ni_3N (**a**), IrO_2 (**b**), Ni_3FeO_x (**c**), and NiO (**d**)

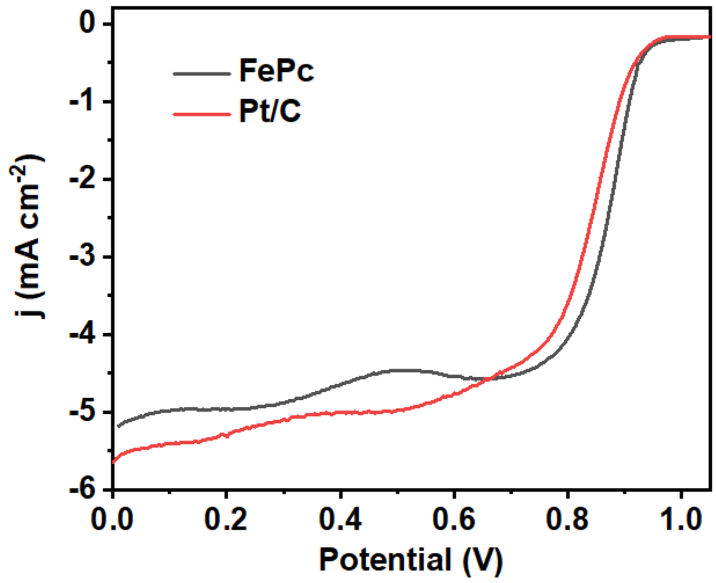

Fig. S10 ORR polarization curves of FePc and Pt/C in O_2-saturated 0.1 M KOH solution

Table S1 Comparison of OER activity of the mesoporous Ni_3FeN with recently reported OER electrocatalysts

Catalysts	Electrolyte	Over-potential at 10 mA cm^{-2} (mV)	Tafel slope mV dec^{-1}	Refs.
**Co-Zn(3:1)-N**	1M KOH	350	75.06	[S8]
**Co-Mn(3:1)-N**	1M KOH	330	73.53	[S8]
**CoNi(3:1)-N**	1M KOH	320	70.45	[S8]
**NiFeN/r-GO**	1 M KOH	270	54	[S9]
**Ni_3FeN Bluk**	1 M KOH	320	67	[S10]
**Ni_3Fe/Ni_3FeN**	1M KOH	268	54	[S11]
**Yolk–Shell Ni_3FeN**	1M KOH	295	69	[S11]
**Co_3FeN_x**	1M KOH	222	46	[S12]
**$NiCo_2N$-NF**	1M KOH	290	65	[S13]
**Cu_{1-x} NNi_{3-y}/FeNiCu**	1M KOH	280	46	[S14]
**TiN@Ni_3N**	1M KOH	350	94	[S15]
**$Ni_{0.75}$ $Fe_{0.25}$ LDH**	1M KOH	350	64	[S16]
**NiFeMn LDH**	1M KOH	270	47	[S17]
**NiFe LDH-UF**	1M KOH	254	32	[S18]
**Porous CoFe–P**	1M KOH	270	30	[S19]
**NiSe**	1M KOH	270	64	[S20]
**CoP/ $CoCr_2O_4$**	1M KOH	290	52	[S21]
**Ir@IrO_2**	1M KOH	255	45	[S22]
**$NiCo_2O_3$@OMC**	1 M KOH	281	96.8	[S23]
**Mesoporous Ni_3FeN**	1 M KOH	259	54	This work

Supplementary References

[S1] E. Rossinyol, J. Arbiol, F. Peiró, A. Cornet, J. R. Morante, B. Tian, D. Zhao, Nanostructured metal oxides synthesized by hard template method for gas sensing applications. Sens. Actuat. B: Chem. **109**, 57-63 (2005). https://doi.org/10.1016/j.snb.2005.03.016

[S2] S. Trasatti, O. A. Petrii, Real surface area measurements in electrochemistry. Pure Appl. Chem. **63**, 711-734 (1991). https://doi.org/10.1351/pac199163050711

[S3] Q. Liu, J. Li, Z. Zhao, M. Gao, L. Kong, J. Liu, Y. Wei, Design, synthesis and catalytic performance of vanadium-incorporated mesoporous silica KIT-6 catalysts for the

oxidative dehydrogenation of propane to propylene. Catal. Sci. Technol. **6**, 5927-5941 (2016). https://doi.org/10.1039/C6CY00404K

[S4] S. Brunauer, P.H. Emmett, E. Teller, "Adsorption of gases in multimolecular layers, J. Am. Chem. Soc. **60,** 309-319 (1938). https://doi.org/10.1021/ja01269a023

[S5] E.P. Barrett, L.G. Joyner, P.P. Halenda, The determination of pore volume and area distributions in porous substances. I. Computations from nitrogen isotherms. J. Am. Chem. Soc. **73**, 373-380 (1951). https://doi.org/10.1021/ja01145a126

[S6] A. Boulaoued, I. Fechete, B. Donnio, M. Bernard, P. Turek, F. Garin, Mo/KIT-6, Fe/KIT-6 and Mo–Fe/KIT-6 as new types of heterogeneous catalysts for the conversion of MCP. Micropor. Mesopor. Mat. **155**, 131- 142 (2012). https://doi.org/10.1016/j.micromeso.2012.01.028

[S7] F. Song, W. Li, J. Yang, G. Han, P. Liao,Y. Sun, Interfacing nickel nitride and nickel boosts both electrocatalytic hydrogen evolution and oxidation reactions. Nat. Commun. **9**, 4531-4540 (2018). https://doi.org/s41467-018-06728-7

[S8] T. Liu, M. Li, X. Bo, M. Zhou, Comparison study toward the influence of the second metals doping on the oxygen evolution activity of cobalt nitrides. ACS Sustain. Chem. Eng. **6**, 11457-11465 (2018). https://doi.org/10.1021/acssuschemeng.8b01510

[S9] Y. Gu, S. Chen, J. Ren, Y.A. Jia, C. Chen, S. Komarneni, D. Yang, X. Yao, Electronic structure tuning in Ni_3FeN/r-GO aerogel toward bifunctional electrocatalyst for overall water splitting. ACS Nano **12**, 245-253 (2018). https://doi.org/10.1021/acsnano.7b05971

[S10] X. Jia, Y. Zhao, G. Chen, L. Shang, R. Shi et al., Ni_3FeN nanoparticles derived from ultrathin NiFe-layered double hydroxide nanosheets: an efficient overall water splitting electrocatalyst. Adv. Energy Mater. **6**, 1502585 (2016). https://doi.org/10.1002/aenm.201502585

[S11] H. Li, S. Ci, M. Zhang, J. Chen, K. Lai, Z. Wen, Facile spray‐pyrolysis synthesis of yolk–shell earth‐abundant elemental nickel–iron‐based nanohybrid electrocatalysts for full water splitting. ChemSusChem **10**, 4756-4763 (2017). https://doi.org/10.1002/cssc.201701521

[S12] Y. Wang, D. Liu, Z. Liu, C. Xie, J. Huo, S. Wang, Porous cobalt–iron nitride nanowires as excellent bifunctional electrocatalysts for overall water splitting. Chem Commun. **52**, 2614-12617 (2016).https://doi.org/10.1039/C6CC06608A

[S13] Y. Wang, B. Zhang, W. Pan, H. Ma, J. Zhang, Porous nickel–cobalt nitrides supported on nickel foam as efficient electrocatalysts for overall water splitting, ChemSusChem **10**, 4170-4177 (2017). https://doi.org/10.1002/cssc.201701456

[S14] Y. Zhu, G. Chen, Y. Zhong, Y. Chen, N. Ma, W. Zhou, Z. Shao, A surface-modified antiperovskite as an electrocatalyst for water oxidation. Nat. Commun. **9**, 2326 (2018). https://doi.org/s41467-018-04682-y

[S15] Q. Zhang, Y. Wang, Y. Wang, A.M. Al-Enizi, A.A. Elzatahry, G. Zheng, Myriophyllum-like hierarchical TiN@ Ni_3N nanowire arrays for bifunctional water splitting catalysts. J. Mater. Chem. A **4**, 5713-5718 (2016). https://doi.org/10.1039/C6TA00356G

[S16] K. Fan, H. Chen, Y. Ji, H. Huang, P.M. Claesson et al., Nickel–vanadium monolayer double hydroxide for efficient electrochemical water oxidation. Nat. Commun. **7**(1), 1-9 (2016). https://www.nature.com/articles/ncomms11981

[S17] Z. Lu, L. Qian, Y. Tian, Y. Li, X. Sun, X. Duan, Ternary NiFeMn layered double hydroxides as highly-efficient oxygen evolution catalysts. Chem. Commun. **52**(5), 908-911 (2016). https://doi.org/10.1039/C5CC08845C

[S18] Y. Zhao, X. Zhang, X. Jia, G.I. Waterhouse, R. Shi et al., Sub-3 nm ultrafine monolayer layered double hydroxide nanosheets for electrochemical water oxidation. Adv. Energy Mater. **8**(18), 1703585 (2018). https://doi.org/abs/10.1002/aenm.201703585

[S19] Y. Tan, H. Wang, P. Liu, Y. Shen, C. Cheng et al., Versatile nanoporous bimetallic phosphides towards electrochemical water splitting. Energy Environ. Sci. **9**(7), 2257-261 (2016). https://doi.org/10.1039/C6EE01109H

[S20] C. Tang, N. Cheng, Z. Pu, W. Xing, X. Sun, NiSe nanowire film supported on nickel foam: an efficient and stable 3D bifunctional electrode for full water splitting. Angew. Chem. Int. Ed. **54**(32), 9351-9355 (2015). https://doi.org/10.1002/anie.201503407

[S21] A. Saad, H. Shen, Z. Cheng, Q. Ju, H. Guo, M. Munir, A. Turak, J. Wang, M. Yang, Three-dimensional mesoporous phosphide-spinel oxide heterojunctions with dual function as catalysts for overall water splitting. ACS Appl. Energy Mater. (2020). https://doi.org/10.1021/acsaem.9b02155

[S22] W. Zhong, Z. Lin, S. Feng, D. Wang, S. Shen et al., Improved oxygen evolution activity of IrO_2 by in situ engineering of an ultra-small Ir sphere shell utilizing a pulsed laser. Nanoscale **11**(10), 4407-4413 (2019). https://doi.org/10.1039/C8NR10163A

[S23] Y. Zhang, X. Wang, F. Luo, Y. Tan, L. Zeng, B. Fang, A. Liu, Rock salt type $NiCo_2O_3$ supported on ordered mesoporous carbon as a highly efficient electrocatalyst for oxygen evolution reaction. Appl. Catal. B: Environ. **256**, 117852 (2019). https://doi.org/10.1016/j.apcatb.2019.117852
